# Invasive Fusariosis in Patients with Hematologic Diseases

**DOI:** 10.3390/jof7100815

**Published:** 2021-09-28

**Authors:** Marcio Nucci, Gloria Barreiros, Tiyomi Akiti, Elias Anaissie, Simone A. Nouér

**Affiliations:** 1 Department of Internal Medicine, Faculdade de Medicina, Universidade Federal do Rio de Janeiro, Rio de Janeiro 21941-913, Brazil; 2 Mycology Laboratory, Hospital Universitário Clementino Fraga Filho, Universidade Federal do Rio de Janeiro, Rio de Janeiro 21941-913, Brazil; mgcb@hucff.ufrj.br (G.B.); akiti@hucff.ufrj.br (T.A.); 3 CTI Clinical Trial & Consulting Services, Cincinnati, OH 41011, USA; eanaissie@ctifacts.com; 4 Department of Infectious Diseases, Faculdade de Medicina, Universidade Federal do Rio de Janeiro, Rio de Janeiro 21941-913, Brazil; snouer@hucff.ufrj.br

**Keywords:** *Fusarium*, fusariosis, fungal infection, fungemia, immunocompromised, neutropenic

## Abstract

*Fusarium* species are filamentous fungi widely encountered in nature, and may cause invasive disease in patients with hematologic conditions. Patients at higher risk are those with acute leukemia receiving induction remission chemotherapy or allogeneic hematopoietic cell transplant recipients. In these hosts, invasive fusariosis presents typically with disseminated disease, fever, metastatic skin lesions, pneumonia, and positive blood cultures. The prognosis is poor and the outcome is largely dependent on the immune status of the host, with virtually a 100% death rate in persistently neutropenic patients, despite monotherapy or combination antifungal therapy. In this paper, we will review the epidemiology, clinical manifestations, diagnosis, and management of invasive fusariosis affecting patients with hematologic diseases.

## 1. Introduction

Invasive fungal disease (IFD) is a serious complication in patients with hematologic malignancies, with the highest incidence occurring in patients with acute leukemia and in hematopoietic cell transplant (HCT) recipients [1,2]. Until the 1980s, yeasts, particularly *Candida* species, were the most frequent agents of IFD. However, with the introduction of fluconazole prophylaxis, IFD caused by molds became more prevalent [3]. While Aspergillus species account for the majority of cases of IFD in hematologic patients, infection caused by other molds, such as *Fusarium* species, may occur, with a relatively high incidence in some areas of the globe [4]. In this article, we will review the epidemiology, clinical manifestations, diagnosis, and management of invasive fusariosis in patients with hematologic diseases.

## 2. The Fungus

*Fusarium* species are ubiquitous filamentous fungi, commonly found in the soil, plants, and water [4]. They are important agents of disease in plants [5] and are part of water biofilms in hospital water systems worldwide [6,7,8,9]. In non-immunocompromised individuals, the most frequent infections caused by *Fusarium* species are onychomycosis and keratitis [10,11]. Immunosuppressed patients with hematologic diseases may develop invasive fusariosis, with disseminated skin lesions, positive blood cultures, and a poor outcome [12].

The genus *Fusarium* comprises more than 300 phylogenetically distinct species, grouped in more than 20 species complexes [13,14]. However, *Fusarium* species causing disease in humans are grouped into seven species complexes (Table 1): *Fusarium solani* species complex (FSSC), *Fusarium oxysporum* species complex (FOSC), *Fusarium fujikuroi* species complex (FFSC), *Fusarium incarnatum-equiseti* species complex (FIESC), *Fusarium chlamidosporum* species complex (FCSC), *Fusarium dimerum* species complex (FDSC), and *Fusarium sporotrichoides* species complex (FSAMSC) [15]. Approximately 70% of cases of invasive disease occurring in hematologic patients are caused by FSSC and FOSC [4], but there are geographic differences in species distribution [16,17,18].

The detection of the growth of *Fusarium* in clinical specimens is not difficult in a routine mycology laboratory. *Fusarium* species grow rapidly on many media without cycloheximide. The colonies in potato dextrose agar are pink, red, gray, or yellow, with velvety to cottony surfaces. The genus is easily identified by the typical banana-shaped macroconidia. However, species identification requires molecular methods [19] or mass spectrometry using matrix-assisted laser desorption–ionization flight time (MALDI-TOF). The latter has been evaluated in both pure colonies and blood culture bottles, and is the easiest method for species identification [19,20]. Adventitious sporulation is characteristic of *Fusarium*, and the yeast-like structures, called aleuroconidia, are responsible for the frequent occurrence of positive blood cultures and metastatic skin lesions [12]. In tissue, the hyphae of *Fusarium* are hyaline, septate with acute-angle branching, with an appearance similar to Aspergillus. Sometimes aleuroconidia are found in tissue together with hyphae, which is suggestive of *Fusarium*. However, since the appearance of these hyaline hyphae in tissue is quite similar among different fungi, the term hyalohyphomycosis is more appropriate when the genus is not identified. This underscores the importance of culture of tissue biopsy, together with histopathology. If culture is not available or does not grow the fungus, in-situ hybridization in paraffin-embedded tissue may help to define the genus [21].

## 3. Epidemiology

### 3.1. Setting

In patients with hematologic diseases, invasive fusariosis occurs more frequently in acute leukemia and in allogeneic HCT. In a series of 38 cases of fusariosis occurring in a 10-year period at a cancer center in the USA, 30 (78.9%) occurred in patients with acute leukemia, being 23 cases in acute myeloid leukemia (AML), and 7 in acute lymphoid leukemia (ALL). The disease occurred in the context of HCT (12 patients) or after chemotherapy (18 patients) [12]. The authors performed a literature review and found 54 additional cases. Again, acute leukemia was the most frequent underlying disease (85.2%, being 26 with AML and 20 with ALL).

In another study of invasive fusariosis in patients with hematologic diseases, we reported 84 cases from 11 centers in Brazil and 1 in the USA. The most frequent underlying diseases were AML (29 cases, 35%), ALL (18 cases, 21%), and chronic myeloid leukemia (CML, 13 cases, 15%). Fusariosis occurred after HCT in 33 patients (39%), including the 13 patients with CML. Other underlying diseases included myelodysplasia, Hodgkin’s and non-Hodgkin’s lymphoma, multiple myeloma, aplastic anemia, and chronic lymphoid leukemia (CLL) [22]. In the largest published series of invasive fusariosis, (233 patients diagnosed in 44 centers from 11 countries), 215 (92%) had a hematologic disease. AML (91 cases) and ALL (46 cases) were the most frequent underlying diseases, and 104 cases occurred after HCT (89 after allogeneic HCT). Other hematologic diseases included CML (22 cases), myelodysplasia (MDS, 13 cases), aplastic anemia (13 cases), non-Hodgkin’s lymphoma (11 cases), multiple myeloma (10 cases), and myelofibrosis (1 case) [23].

An emerging setting for IFD in hematology is represented by patients with chronic lymphoproliferative diseases (CLPDs) (especially CLL) receiving ibrutinib. In addition to its effect in inhibition of Bruton kinase, ibrutinib inhibits various components of the immune system; in fact, it is approved for the treatment of corticosteroid-refractory chronic graft versus host disease (GVHD) [24]. Epidemiologic studies reported an increase in the incidence IFD in patients with CLPDs receiving ibrutinib, especially invasive aspergillosis (IA) [25]. However, other IFDs, including invasive fusariosis, have also been diagnosed in such patients [26,27].

In most patients with hematologic conditions and invasive fusariosis, the infection develops in the setting of neutropenia. In a study of 58 cases of invasive fusariosis in Spanish centers, only 24 (24%) occurred in patients without neutropenia. Among the 44 neutropenic patients, 41 (93.2%) had a hematologic condition compared to 5 of 14 (35.7%) among those who were non-neutropenic. In three of the latter five patients, the infection developed after allogeneic HCT given for a hematologic disease [28].

In patients with acute leukemia, invasive fusariosis usually develops in patients with uncontrolled disease, and virtually all such patients are neutropenic. In our series of 84 patients with fusariosis, only 15% were in complete hematologic remission, and 83% were neutropenic, with a median duration of 16 days before the diagnosis of fusariosis (range, 2–93 days) [22]. In a more recent series published by our group, 85% of 26 patients with fusariosis were neutropenic, with a median duration of neutropenia before the diagnosis of fusariosis of 11 days (range 153).

By contrast, invasive fusariosis among allogeneic HCT recipients develops in patients with adequate neutrophil count. These patients typically have severe T-cell immunodeficiency caused by GVHD and its treatment. Among 54 allogeneic HCT recipients, invasive fusariosis has a trimodal distribution; a first peak early during neutropenia (median of 16 days post-HCT), a second peak between days 61 and 80 post-HCT (median, 64 days), while the third peak develops after day 360. The second and third peaks occurred in the context of acute and chronic GVHD, respectively, and most patients were not neutropenic [29].

### 3.2. Incidence and Risk Factors

As shown in Table 2, the incidence of invasive fusariosis in patients with hematologic diseases varies across different regions of the globe with the highest incidence reported in Brazil, compared with other countries such as Italy, USA, and Spain [1,12,28,29,30,31,32,33,34,35].

The risk factors for invasive fusariosis were evaluated in a cohort of patients with AML or MDS receiving intensive induction chemotherapy, and among HCT recipients. The only factor identified in the AML/MDS cohort was active smoking, with a hazard ratio (HR) of 9.11 (95% confidence interval [95% CI] 2.04–40.71). Among allogeneic HCT recipients, variables associated with invasive fusariosis diagnosed in the pre–engraftment period were the receipt of antithymocyte globulin (HR 22.77, 95% CI 4.85–101.34), hyperglycemia (HR 5.17, 95% CI 1.40–19.11), treatment in one of the participating centers (HR 5.15, 95% CI 1.66–15.97) and AML as underlying disease (HR 4.38, 95% CI 1.39–13.81). Risk factors in the post–engraftment period were non–myeloablative conditioning regimen (HR 35.08, 95% CI 3.90–315.27), grade III–IV GVHD (HR 16.50, 95% CI 2.67–102.28), and a history of invasive mold disease (HR 10.65, 95% CI 1.19–95.39) [36].

### 3.3. Mode of Acquisition

The airways are the most frequent portals of entry of *Fusarium* species. This is supported by the presence of airborne fusarial conidia, and the fact that the airways (sinuses and lungs) are the most commonly affected sites [4,37]. The skin at sites of tissue breakdown may also be a portal of entry for both locally invasive disease, such as cellulitis, as well as disseminated disease. Other possible portals of entry are the gastrointestinal tract and central venous catheters [4,38]. Onychomycosis is the most frequent pre-existing superficial lesion predisposing to disseminated disease [39]. A patient with hematologic disease and onychomycosis may subsequently develop cellulitis on the toe of the diseased nail, followed by disseminated fusariosis, with metastatic skin lesions. Interdigital intertrigo is another superficial skin lesion that predisposes to invasive fusariosis [40].

*Fusarium* species are present in the outdoor and indoor air [8,9,38,41,42]. Therefore, invasive fusariosis may be acquired both in the community and in the hospital. In the hospital, invasive fusariosis may be acquired by inhalation of primarily contaminated air, as reported in outbreak investigations [41,42]. In addition, the disease may be acquired from contaminated water, either by direct contact of a damaged skin, or by inhalation of aerosols. *Fusarium* species are frequently recovered from hospital water cultures [8,9], and showering and other water-related activities appear to be an efficient mechanism for the dispersion of airborne fusarial conidia [43].

## 4. Clinical Manifestations

The epidemiology and clinical picture of invasive fusariosis share similarity with those of invasive aspergillosis. However, there are important differences (Table 3).

### 4.1. The Four Most Common Clinical Presentations

The four most common clinical presentations of invasive fusariosis are (a) fever and metastatic skin lesions; (b) pneumonia; (c) fever and positive blood culture for a mold; and (d) cellulitis or lymphangitis at sites of skin breakdown [22,44]. Less frequently, patients may present with single organ involvement, such as arthritis and sinusitis [45,46], or catheter-related fungemia [38,47].

### 4.2. Skin Lesions

Skin lesions are frequent in invasive fusariosis and are usually part of disseminated disease. We have previously characterized the cutaneous manifestations of invasive fusariosis analyzing 259 cases, 232 of which occurred in immunosuppressed patients [48]. Skin lesions were present in 72% of immunocompromised patients, mostly disseminated. Eleven of the twenty cases with localized skin lesions had a history of tissue breakdown, especially onychomycosis with periungueal cellulitis. The most frequent lesions of disseminated fusariosis were papules and nodules with or without necrosis. Lesions at different stages of evolution were frequent, and many patients complained of myalgia. The skin is frequently the single source of diagnosis and a biopsy of one of such lesions is the fastest way of establishing the diagnosis of invasive fusariosis (see below) [49].

As mentioned above, many hematologic patients with invasive fusariosis present with skin breakdowns in the lower extremities with subsequent development of cellulitis or lymphangitis, and may evolve to disseminated disease. We have previously reported an increase in the incidence of invasive fusariosis with a cutaneous portal of entry in our institution. Among 21 cases diagnosed in an 11-year period, a cutaneous portal of entry (onychomycosis and or interdigital intertrigo) was present in 11 [50].

### 4.3. Pneumonia

Pneumonia is a frequent manifestation of invasive fusariosis and may occur as single-organ involvement or, more frequently, as part of disseminated disease. In a review of 317 cases of invasive fusariosis in immunocompromised patients, pneumonia was noted in 145 patients (46%), most of which was bilateral (73%) [37]. In another study, among 233 cases of invasive fusariosis, pneumonia was present in 114 cases, with 105 cases occurring as part of disseminated fusariosis, and 9 as isolated pneumonia [23].

In neutropenic patients, the pathogenesis of pneumonia by *Fusarium* species acquired by the airways is similar to that of invasive aspergillosis, with inhalation of airborne conidia, colonization of the alveoli, with subsequent development of hyphae, bronchoalveolar involvement and dissemination, and angioinvasion with lung infarction. However, unlike aspergillosis in which the airways are the portal of entry in virtually all cases, lung involvement by *Fusarium* species may also have a cutaneous portal of entry, with subsequent hematogenous dissemination to the lungs. In an analysis of 105 cases of invasive fusariosis in which pneumonia was part of disseminated disease, bilateral lung involvement was more frequent among patients with a cutaneous portal of entry (88%) than among those who had a non-cutaneous portal of entry (68%) [37].

The radiologic picture of pulmonary fusariosis on chest computed tomography (CT) scans was described in 11 cases. Nodules which were less than 3 cm in size were present in nine cases, two of which had a halo of ground-grass infiltrates (halo sign). Other frequent images were nodules larger than 3 cm (six cases, one of which with cavitation) and consolidations (four cases) [51]. In another study, chest CT imaging of 9 cases of invasive fusariosis were compared with the images of 11 cases of aspergillosis and three of mucormycosis. Images of bronchoalveolar involvement and dissemination (centrilobular micronodules, tree-in-bud infiltrates) were more frequent in fusariosis (7 of 9, 78%) compared with aspergillosis (1 of 11, 9%) and mucormycosis (no case). Peribronchial consolidations and air bronchogram were also more frequent in fusariosis (56% vs. 9% vs 0 in fusariosis, aspergillosis, and mucormycosis, respectively). By contrast, macronodules were more frequent in aspergillosis (100%) and mucormycosis (100%) compared with fusariosis (44%). Notably, none of the four patients with fusariosis presenting with macronodules had a halo sign [52].

We compared the radiologic pattern of 13 cases of invasive fusariosis with 32 cases of aspergillosis. We did not find a statistically significant difference in the frequency of macronodules (61.5% vs. 78.1%), centrilobular micronodules (58.3% vs. 43.8%), tree-in-bud (7.7% vs. 12.5%), or ground-grass infiltrates (53.8% vs. 43.8%). By contrast, cases of fusariosis were less likely to have consolidations (23.1% vs. 50.0%, *p* = 0.10), cavity (0 vs. 21.9%, *p* = 0.09), and nodules with the halo sign (23.1% vs. 62.5%, *p* = 0.02) [44]. 

We must acknowledge that these comparisons have important limitations because the predominant radiologic pattern depends on the time in which images were obtained in relation to the initiation of the disease process and the patient’s immune status [53]. Taking the model as an invasive mold disease acquired by inhalation of conidia, the first phase of the disease is characterized by bronchoalveolar involvement and dissemination. In this phase, ground-grass infiltrates, centrilobular micronodules, and tree-in-bud infiltrates predominate. As the disease advances, angioinvasion occurs and the radiologic picture is dominated by the appearance of macronodules with a halo sign. However, if a chest CT scan is performed a few days later, the halo sign disappears, and non-specific alveolar consolidations predominate [54]. Air crescent lesions and cavities may appear coinciding with subsequent neutrophil recovery [53]. On the basis on these findings, the radiologic pattern is unlikely to distinguish between fusariosis and aspergillosis.

### 4.4. Sinusitis

Sinusitis is a frequent manifestation of invasive fusariosis in patients with hematologic diseases. Among 233 cases of invasive fusariosis from 40 centers worldwide, sinusitis was present in 72 cases (31%) [23]. In a review of 262 cases of invasive fusariosis diagnosed in immunocompromised patients, sinusitis was reported in 52 (20%). In hematologic patients, sinusitis occurred in the context of disseminated disease in 70% of cases, and was the source of diagnosis in 11% of all cases [22,46].

Many hematologic patients with sinusitis by *Fusarium* species do not have symptoms, and sinusitis is diagnosed on the basis of CT scan imaging. Clinical manifestations include nasal discharge, obstruction, and necrotic lesions. In advanced disease, periorbital and paranasal cellulitis may occur. In our study, sinusitis was more frequent among patients with aspergillosis than those with fusariosis (63.9% vs. 38.5%, *p* = 0.048). The radiologic manifestations of fusarial sinusitis were mucosal thickening (100%), opacity (60%), and air fluid level (10%) [44].

### 4.5. Fungemia

Fungemia is a common manifestation of invasive fusariosis, and usually occurs in the context of disseminated disease. Among 84 cases of invasive fusariosis in patients with hematologic diseases, fungemia occurred in 46 (55%) [22], and in 37% of 233 cases diagnosed in 40 centers worldwide [23]. The reason why fungemia is frequent in invasive fusariosis is because *Fusarium* species produce yeast-like structures, called aleuroconidia, which invade the bloodstream [4].

Occasionally, fungemia is the only manifestation of fusarial infection, usually in non-neutropenic patients with a central venous catheter, and the catheter is likely the source of fungemia. Antifungal treatment and catheter removal result in the cure of fungemia in most cases [38,47].

### 4.6. Disseminated Infection

Disseminated infection is the most frequent clinical presentation of invasive fusariosis, occurring in 79% of 84 patients with hematologic diseases [22], and 75% among 61 HCT recipients [29]. Typically, patients have a combination of metastatic skin lesions, pneumonia, and positive blood cultures. Eye involvement may occur as a form of endophthalmitis or blindness secondary to thrombosis of retinal vessels [12,55]. Disseminated fusariosis is a reflection of severe immunosuppression and a very high fungal burden, and is associated with poor outcome [23].

### 4.7. Other Clinical Manifestations

Occasionally, patients with hematologic diseases present with involvement of other organs such as joints and bones. In a literature review of bone and joint infections caused by molds, five cases caused by *Fusarium* species were reported, one of which occurred in a patient with AML, in the context of disseminated fusariosis [56]. A case of arthritis caused by *Fusarium* solani was reported in an allogeneic HCT recipient as a complication of disseminated fusariosis [57]. We have seen a few cases of arthritis after allogeneic HCT, all occurring as a late complication of disseminated disease in the setting of severe immunosuppression. Typically, the patients have disseminated disease with multiple skin lesions and positive blood cultures, improve with neutrophil recovery and antifungal treatment, and one to three weeks later present with unilateral joint swelling. Aspiration of the joint fluid usually grows *Fusarium* species.

## 5. Diagnosis of Invasive Fusariosis

The confirmation of the diagnosis of invasive fusariosis depends on the growth of the organism in culture of biologic materials and/or the demonstration of tissue invasion by hyphae. As mentioned above, the sole demonstration of septate, acute-branching, and hyaline hypha in tissue is not enough to establish the diagnosis of invasive fusariosis as other hyaline molds have the same histopathologic picture. In such circumstances, the most appropriate diagnosis is hyalohyphomycosis.

The most frequent sources of diagnosis are the blood and skin biopsy. Among 84 hematologic patients with invasive fusariosis, the diagnosis was made by culture in 65 patients (77.4%): blood culture (26 cases), culture of a fragment of skin biopsy (18 cases), culture of blood and skin biopsy (12 cases), culture of sinus tract (eight cases), and culture of a bronchoalveolar lavage (BAL) fluid (one case). In the remaining 19 cases, the diagnosis was made by culture and histopathology (blood and skin in 18, and sinus in one) [22]. In another series with 233 cases of invasive fusariosis, detailed information about the diagnosis was available in 224 cases: culture alone in 138 (61.6%), culture and histopathology in 83 (37.0%), and histopathology alone in three. The skin was the main source of diagnosis in 100 cases, followed by blood (85 cases) [23].

A few studies evaluated the performance of different blood culture systems or bottles. The performance of a specific fungal medium and a standard aerobic medium of Bactec™ were compared, with better performance of the fungal medium [58]. In another study, bacteria and molds (including *Fusarium* species) were inoculated in the same blood culture bottles of Bactec™. Bottles with selective fungal media had a better performance than aerobic media [59]. In our experience with BacTAlert™ aerobic bottles, the median time to positivity of blood cultures growing *Fusarium* species was three days (range 1–4) [49].

In patients with multiple skin lesions, it is critical to appropriately select one lesion for biopsy. Preference should be given to nodular painful lesions, or lesions with an echthyma gangrenosum appearance. The biopsy should be deep enough to reach blood vessels in the deep dermis, and to see thrombosis of the vessels with hyaline hyphae within the vessels. Part of the fragment of skin should be sent to histopathology and part to direct exam and culture [48].

Direct microscopic exam of biologic material is an important component of the diagnosis of invasive fusariosis. We analyzed the time taken to diagnose invasive fusariosis in 18 patients. The fastest way of reaching a presumptive diagnosis was by a direct exam of a skin biopsy (two patients) or other biological material (sinus aspirate and BAL, one patient each). The finding of hyaline hyphae in the direct exam was reached a few hours after the procedure, and prompted the immediate initiation of antifungal treatment. By contrast, the median time to positivity of blood culture was three days (range 1–4) [49].

Although considered specific for aspergillosis, serum galactomannan, as detected by the Platelia Aspergillus enzyme immunoassay (BioRad), may be positive in infection caused by other fungi, including *Fusarium* species [60]. We evaluated the performance of serum galactomannan in hematologic patients with invasive fusariosis diagnosed in three centers in Brazil. Among 18 patients, 15 (83%) had at least one positive serum galactomannan test (median of 4 positive tests). Serum galactomannan was positive before the first clinical manifestation of invasive fusariosis in 8 patients, and in 11 before the diagnosis of fusariosis [49]. In other study, we compared the characteristics of 36 patients with invasive aspergillosis with 26 patients with invasive fusariosis. Serum galactomannan was positive in 88.6% and 73.3% of patients with aspergillosis and fusariosis, respectively, with no differences in the median number of positive tests and galactomannan values [44]. Therefore, in regions where invasive fusariosis is more frequent, patients with lung nodules and positive serum galactomannan may have either aspergillosis or fusariosis.

Another fungal biomarker that has potential in the diagnosis of invasive fusariosis is serum 1,3-β-D-glucan. We evaluated the performance of 1,3-β-D-glucan in 13 patients with invasive fusariosis. Twelve of the thirteen patients (92.3%) had at least one positive 1,3-β-D-glucan serum level (median of four). The test was positive before the diagnosis of invasive fusariosis in 11 of the 12 patients, at a median of 10 days. Comparing this group with a control group of hematologic patients with similar underlying diseases and treatments, the sensitivity, specificity, and positive and negative predictive values of two consecutive positive beta-glucan tests were 85%, 69%, 7%, and 99%, respectively. We concluded that, while the test is positive in the majority of patients with invasive fusariosis, the low positive predictive value strongly limits its usefulness in the diagnosis [60].

## 6. Management of Invasive Fusariosis

### 6.1. Antifungal Susceptibility

*Fusarium* species exhibit high minimum inhibitory concentrations (MICs) to almost all antifungal drugs. In general, the MICs are higher for the azoles voriconazole and posaconazole compared with amphotericin B, and higher for FSSC compared with FOSC isolates. In a multicenter study, 1150 isolates belonging to different *Fusarium* species complexes were tested against various antifungal agents in order to establish epidemiologic cutoff values. For FSSC (608 isolates), MIC ranges were ≤0.25–16 µg/mL for amphotericin B, 0.5–>16 µg/mL for voriconazole, and 1–>16 µg/mL for posaconazole. For FOSC, MIC ranges were ≤0.25–16 µg/mL for amphotericin B, 0.5–>16 µg/mL for voriconazole, and 0.5–>16 µg/mL for posaconazole [61].

The activity of isavuconazole against 14 *Fusarium* species isolates (including 7 FSSC and 6 FFSC) was evaluated. The MIC_50_ was >4 µg/mL (range 2–>8). Using the same isolates, the MIC_50_ of voriconazole was 8 µg/mL (range 2–>8) [62]. In other study, the susceptibility to isavuconazole was tested in 75 clinical isolates. The MIC_50_ against 31 FFSC, 22 FSSC, and 17 FOSC isolates was >16 µg/mL (range 4–>16), >16 µg/mL (range 4–>16) and 8 µg/mL (range 2–>16), respectively [63].

The in vitro activity of two new antifungal agents was evaluated against *Fusarium* species isolates. The first is olorofim, an agent belonging to the orotomide drug class. The MIC_50_ against 45 isolates of FOSC and 16 isolates of FSSC was 0.5 µg/mL (range 0.06–>4) and >4 µg/mL (range 1–>4), respectively. Tested against the same isolates, the MIC_50_ for voriconazole and amphotericin B were 8 µg/mL (range 4–16) and 2 µg/mL (range 1–4), respectively, against FOSC, and >16 µg/mL (range 2–>16) and 1 µg/mL (range 0.25–4), respectively, against FSSC [64]. The other agent is fosmanogepix (active compound manogepix). The MIC range against 49 FOSC and 19 FSSC isolates was ≤0.015–0.125 µg/mL and ≤0.015–0.25 µg/mL, respectively. As a comparison, the MIC ranges against voriconazole were 4–16 µg/mL for FOSC, and 2–>16 µg/mL for FSSC [65].

The key question regarding antifungal susceptibility in invasive fusariosis is centred around the extent to which the results of in vitro activity may help to select the appropriate treatment. In other words, what is the correlation between MIC and outcome? No correlation between MIC and outcome was observed in an in vivo murine model of invasive fusariosis [66]. We evaluated the correlation between MIC and clinical outcome in 88 cases of invasive fusariosis, 74 of which occurred in patients with hematologic diseases. Among 22 patients treated with voriconazole monotherapy, the MIC_50_ among patients who survived and died were 4 µg/mL and 8 µg/mL, respectively (*p* = 0.68). Likewise, no correlation between MIC and clinical outcome was observed among 21 patients treated with amphotericin B alone or in combination with voriconazole (29 patients) [67]. The lack of correlation between MIC and outcomes is in agreement with a study that showed similar response rates with voriconazole and amphotericin B, despite the higher MICs for voriconazole observed in antifungal susceptibility tests [23]. Furthermore, there are no established breakpoints for *Fusarium* species. These data indicate that in vitro susceptibility tests should not be used to guide the choice for primary treatment of invasive fusariosis. The same is true for species identification. Therefore, species identification and antifungal susceptibility tests are of help for epidemiologic purposes but not to define the primary treatment for invasive fusariosis.

### 6.2. Prognostic Factors

In contrast with the lack of correlation between antifungal susceptibility tests and the outcome, there is a close relationship between immunity and survival. Analyzing prognostic factors in 84 hematologic patients with invasive fusariosis, the 90-day probability of survival was 0% if patients had persistent neutropenia and were receiving corticosteroids, 4% in those with persistent neutropenia only, 30% in patients receiving corticosteroids but not neutropenic, and 67% in patients without any of these two factors [22]. In our analysis of the outcome of 233 cases of invasive fusariosis (215 with hematologic diseases), variables associated with poor outcome (90-day mortality) were again receipt of corticosteroids (HR 2.11), neutropenia at the end of treatment (HR 2.70), and primary treatment with deoxycholate amphotericin B (HR 1.83) [23].

### 6.3. Primary Prophylaxis

Primary anti-mold prophylaxis is usually indicated in hematologic patients at high risk to develop invasive fusariosis, including AML in induction remission [68] and allogeneic HCT [69]. In patients receiving anti-mold prophylaxis, breakthrough infection may occur, including fusariosis [70,71,72].

We evaluated the usefulness of primary prophylaxis in a subset of patients at risk. We observed an increase in the incidence of invasive fusariosis with a cutaneous portal of entry in our institution, with most patients presenting onychomycosis and interdigital intertrigo [50]. Subsequently, in a prospective study, we observed that patients with superficial skin lesions in the feet (interdigital intertrigo and/or onychomycosis) on hospital admission with positive culture for *Fusarium* spp. were at an increased risk to develop invasive fusariosis [40]. We then decided to give primary anti-mold prophylaxis (voriconazole or posaconazole) to patients with these characteristics and compared with patients not receiving primary prophylaxis. Among 20 patients receiving primary prophylaxis, invasive fusariosis occurred in 5.9%, compared with 5.0% among 219 patients not on anti-mold prophylaxis. However, four of five patients with superficial skin lesions with positive cultures for *Fusarium* species who did not received anti-mold prophylaxis developed invasive fusariosis vs. none of six with anti-mold prophylaxis (*p* = 0.01) [73]. On the basis of these data, we recommend: (a) a thorough dermatologic examination of the extremities on admission; (b) culture of any suspicious lesion; and (c) primary prophylaxis with an anti-mold azole (voriconazole or posaconazole) if cultures are positive for *Fusarium* species.

### 6.4. Secondary Prophylaxis

Patients with a prior history of invasive mold disease and who will subsequently be exposed to a period of immunosuppression may theoretically be at risk to present with recurrence of the fungal infection. The use of secondary prophylaxis in such circumstances has been well established in invasive aspergillosis [74,75,76], but there is limited data in other mold infections. We evaluated the usefulness of secondary prophylaxis for invasive fusariosis in a multicenter retrospective study of 40 patients who were successfully treated for invasive fusariosis and were exposed to subsequent periods of immunosuppression (neutropenia in 35 and graft versus host disease in 5). Relapse of invasive fusariosis occurred in 2 of 8 patients (25%) who were not on prophylaxis and in 3 of 32 (9.4%) who received secondary prophylaxis (mostly voriconazole). Considering only patients who had prior disseminated fusariosis, relapse occurred in 2 of 2 (100%) not on secondary prophylaxis and in 3 of 26 (11.5%) who received secondary prophylaxis (*p* = 0.03) [77]. In light of these data, we believe that secondary prophylaxis (voriconazole or a lipid preparation of amphotericin B) should be strongly considered in patients with prior invasive fusariosis who will be exposed to subsequent periods of immunosuppression, especially if the disease was disseminated.

### 6.5. Other Preventive Measures

General preventive measures in hematologic patients at risk of developing invasive fusariosis are focused on preventing patient exposure to sources of *Fusarium* species. These include placing patients in rooms with HEPA filter and positive pressure, and avoiding contact with potentially contaminated water and activities associated with skin breakdown. For elective immunosuppressive therapies, such as HCT, careful examination of the extremities should be undertaken, looking for lesions associated with the subsequent development of invasive fusariosis, such as onychomycosis, interdigital intertrigo, and paronychia [4,40]. Once present, these lesions should be treated before commencing immunosuppressive therapies, if possible. This is particularly difficult in patients with onychomycosis by *Fusarium* species, since these mycoses need long periods of treatment.

### 6.6. Primary Therapy

In the planning for the treatment of invasive fusariosis, three important aspects should be taken into consideration: (a) species identification and antifungal susceptibility tests should not be used as a guide to select the appropriate primary treatment; (b) recovery of host defenses are the key determinants of the outcome; and (c) there are no randomized trials evaluating the optimal regimen for primary therapy.

The largest series published to date analyzed the outcome of 233 patients with invasive fusariosis from 44 centers of 11 countries, diagnosed between 1985 and 2011. A total of 206 patients (88%) received treatment. The most frequent agent voriconazole (16%) and a lipid preparation of amphotericin B (15%). Combination therapy was used in 21 patients. Comparing the period of 1985–2000 (period 1) with 2001–2011 (period 2), the 90-day probability of survival was significantly higher in period 2: 43% vs. 22% (*p* < 0.001). Analyzing only the second period, the 90-day probability of survival was 60% in patients treated with voriconazole, 53% with a lipid formulation of amphotericin B, and 28% with deoxycholate amphotericin B (*p* = 0.04) [23]. On the basis of these data, recently published guidelines recommend either voriconazole or a lipid formulation of amphotericin B as primary treatment for invasive fusariosis [78]. We have used voriconazole at the standard recommended dose of 6 mg/kg/dose every 12 h on day 1 intravenously, followed by 4 mg/kg 12/12 h from day 2 on, switching to the oral preparation (600 mg/d) once the patient is clinically stable. For liposomal amphotericin B and amphotericin B lipid complex, we used the doses of 3 mg/kg/d and 5 mg/kg/d intravenously, respectively. There are no data indicating that higher doses work better. Likewise, there are no data indicating that combination therapy is better than monotherapy, and we particularly do not recommend combination therapy. Nevertheless, recently published guidelines give equal strength of recommendation for monotherapy and combination therapy. The arguments justifying combination therapy are the severity of invasive fusariosis, the uncertainties about adequate serum levels of voriconazole, and the high MICs of *Fusarium* species against voriconazole and amphotericin B [78].

Other agents, such as posaconazole, isavuconazole, and terbinafine, have been used for the primary treatment of invasive fusariosis, either as monotherapy or in combination with other agents [45,79]. Considering the limited experience with these drugs, their role in the treatmet of invasive fusariosis in patients with hematologic diseases is yet to be established.

### 6.7. Assessment of Clinical Response

In patients with disseminated fusariosis, fever, skin lesions, pneumonia, and positive blood culture, we rely on objective parameters to early evaluate progression of the disease. Parameters that indicate no response to treatment include a progressive increase in the number of skin lesions, persistently positive blood cultures, the appearance of new symptoms indicating extension of the disease (e.g., neurologic symptoms, blindness), and worsening respiratory symptoms and images in persistently neutropenic patients. The latter should be judged carefully because, coinciding with neutrophil recovery, clinical symptoms may worsen and lung infiltrates may increase in size [80].

### 6.8. Salvage Therapy

Considering that the outcome of invasive fusariosis is largely dependent on the recovery of host immunity, it is difficult to evaluate if failure was due to inappropriate choice of the antifungal regimen or persistent immunosuppression. The largest series of salvage therapy in invasive fusariosis reported 57 patients treated with voriconazole, with 47% response rate [17]. In other study, the response rate of 21 patients treated with posaconazole was 48% [81].

In our experience of the treatment of invasive fusariosis with monotherapy, if a patient presents with any sign of no response to treatment, as detailed above, we add a second antifungal drug (voriconazole if treatment was started with a lipid formulation of amphotericin B or vice-versa). In patients treated with combination therapy upfront, the addition of other drugs or regimens is unlikely to result in a significant benefit.

### 6.9. Ancillary Treatment Measures

Ancillary therapies include the removal of central venous catheters in cases of catheter-related fungemia [38], and the use of growth factors (granulocyte or granulocyte-monocyte colony-stimulating factors, interferon gamma, and granulocyte transfusions to persistently neutropenic patients). The effect of granulocyte transfusions was evaluated in 11 patients with invasive fusariosis. With a median of seven transfusions per patient, neutrophil counts remained >500/mm^3^ for 36 h in 61% of transfusions. A clinical response was observed in 10 patients, 8 of whom were alive three months after the diagnosis of fusariosis. A literature review of 23 published cases showed a response rate of 30% only [82].

### 6.10. Adherence to Guidelines Recommendations

Recently, the European Confederation of Medical Mycology (ECMM) together with the International Society for Human and Animal Mycology (ISHAM) and the American Society for Microbiology (ASM) published guidelines for the management of rare moulds, including fusariosis, containing recommendations for the diagnosis, prevention and treatment of fusariosis [78]. We developed an 18-item tool that gathers the most relevant recommendations from these guidelines, and may help clinicians and institutions to quantify the quality of care of immunocompromised patients who develop invasive fusariosis [83].

## 7. Conclusions

Invasive fusariosis is a serious fungal disease affecting high-risk hematologic patients, especially AML patients receiving induction remission chemotherapy and allogeneic HCT recipients. The most frequent clinical presentation is disseminated disease, with fever, metastatic skin lesions, pneumonia, and positive blood cultures. The outcome is largely dependent on recovery of host defenses, with virtually a 100% death rate in persistently neutropenic patients, despite monotherapy or combination antifungal therapy. The key points for the management of invasive fusariosis are summarized in Table 4.

## Figures and Tables

**Table 1:** Most frequent species complexes of the genus *Fusarium* involved in human infections and their respective species within each complex.

Species Complex	Species Complex
*Fusarium solani* species complex	*Fusarium fujikuroi* species complex
* Fusarium falciforme*	*Fusarium acutatum*
* Fusarium keratoplasticum*	*Fusarium anthophilum*
*Fusarium lichenicola*	*Fusarium andiyazi*
*Fusarium petroliphilum*	*Fusarium fujikuroi*
*Fusarium pseudensiforme*	*Fusarium nygamai*
	*Fusarium proliferatum*
	*Fusarium verticillioides*
*Fusarium oxysporum* species complex	*Fusarium incarnatum-equiseti* species complex
*Fusarium oxysporum*	*Fusarium incarnatum*
*Unnamed*	*Fusarium equiseti*
	*Unnamed*
*Fusarium sporotrichoides* species complex	*Fusarium dimerum* species complex
*Fusarium aermeniacum*	*Fusarium dimerum*
*Fusarium brachygibbosum*	*Fusarium delphinoides*
*Fusarium langsethiae*	*Fusarium penzigii*
*Fusarium sporotrichioides*	
*Fusarium chlamidosporum* species complex	
* Fusarium chlamidosporum*	

**Table 2:** Incidence of invasive fusariosis in different regions of the globe.

Country	Setting	Number of Patients (Denominator)	Number of Cases	Incidence
Italy [30]	Adult patients with hematologic diseases	351 episodes of infection by molds	6	1.7%
Italy [1]	Adult patients with hematologic malignancies	11,802 patients at risk	15	0.1%
Italy [31]	Adult patients undergoing HCT	3228 patients at risk	3	0.1%; 0.2% in allogeneic and no case in autologous HCT
USA [12]	Adult patients undergoing HCT	1607 patients at risk	12	0.7%; 1.2% in allogeneic and 0.2% in autologous HCT
USA [32]	Cancer patients	Not reported	44	0.04 cases per 1000 patients-day in 1998 and 0.012 cases per 1000 patients-day in 2007–2008
Spain [28]	Hospitalized patients	Not reported	58	0.55 cases per 100,000 admissions
USA and Brazil [29]	HCT recipients (adults and children)	Not reported	61	Cases per 1000 HCT: 5.97 overall; 6.18 in Brazil, 5.89 in the USA; 4.21–5.0 in MRD, 2.28 in HLA-compatible MUD, 20.19 in MMRD, 1.4–2.0 in autologous
Brazil [33]	Adults and children with AML/MDS or HCT	937	23	1-year cumulative incidence: 5.2% in allogeneic HCT, 3.8% in AML/MDS, 0.6% in autologous HCT
Brazil [34]	Adults and children with AML/MDS, ALL or HCT	192	3	1.6% overall; 4.3% in AML/MDS, 2.0% in autologous HCT
Brazil [35]	Adult patients with hematologic diseases	980	17	1.7% overall; 3.1% in allogeneic HCT; 3.1% in acute leukemia

HCT = hematopoietic cell transplantation; MRD = matched-related donor; MUD = matched-unrelated donor; MMRD = mismatched-related donor; AML = acute myeloid leukemia; MDS = myelodysplasia; ALL = acute lymphoid leukemia.

**Table 3:** Similarities and differences between invasive fusariosis and invasive aspergillosis.

	Fusariosis	Aspergillosis
Most common setting	Acute leukemia, induction remission and allogeneic HCT ^1^	Acute leukemia, induction remission and allogeneic HCT
Mode of acquisition	Airways and skin at sites of breakdown	Airways
Hospital reservoirs	Air and water	Air and water
Clinical manifestations [44]		
Fever	Yes, 96%	Yes, 64%
Pneumonia	Yes, 50%	Yes, 89%
Nodules with halo sign	Yes, 23%	Yes, 62%
Centrilobular micronodules	Yes, 54%	Yes, 44%
Tree-in-bud infiltrates	Yes, 8%	Yes, 12%
Sinusitis	Yes, 38%	Yes, 64%
Skin lesions	Yes, 73%	No
Positive blood cultures	Frequent	Rare
Positive serum galactomannan [44]	Yes, 73%	Yes, 89%
Positive 1,3-beta-D-glucan	Yes	Yes

^1^ HCT = hematopoietic cell transplantation.

**Table 4:** Key points in invasive fusariosis.

Key Points
Epidemiology
In acute leukemia: after induction remission, in the setting of severe neutropenia
In allogeneic HCT: pre-engraftment period in the setting of severe neutropenia or after engraftment, in non-neutropenic patients with severe GVHD and receipt of corticosteroids
Acquisition by inhalation of conidia, or from skin breakdown (onychomycosis, interdigital intertrigo, paronychia)
Clinical presentation
Fever and metastatic skin lesions
Pneumonia (radiologic picture indistinguishable from aspergillosis)
Fever and positive blood culture for a mold
Cellulitis or lymphangitis at sites of skin breakdown
Diagnosis
Direct exam of a skin biopsy is the fastest way of establishing the diagnosis
Skin biopsy should be cut in two pieces, one for direct exam and culture and other for histopathology
Blood cultures are frequently positive
Serum galactomannan is frequently positive
Management
Primary prophylaxis should be given to patients with skin lesions in the extremities with positive culture for *Fusarium* species
Secondary prophylaxis should be given to patients who respond to treatment and will be submitted to subsequent periods of immunosuppression
Species identification should not be used to select primary therapy
Antifungal susceptibility tests should not be used to select primary therapy
Primary therapy: lipid formulation of amphotericin B or voriconazole
Recovery of host immunity is the key determinant of the outcome

HCT = hematopoietic cell transplantation; GVHD = graft versus host disease.

## Data Availability

Not applicable.
